# Antineutrophil cytoplasmic antibody-associated vasculitis with predominant truncal muscle weakness: a retrospective case series

**DOI:** 10.3389/fneur.2023.1277337

**Published:** 2023-10-12

**Authors:** Joe Nemoto, Hideaki Nishihara, Taro Yasuhi, Ryota Sato, Michiaki Koga, Takashi Kanda, Masayuki Nakamori

**Affiliations:** ^1^Department of Neurology and Clinical Neuroscience, Yamaguchi University Graduate School of Medicine, Yamaguchi, Japan; ^2^Department of Neurotherapeutics, Yamaguchi University of Medicine, Yamaguchi, Japan; ^3^Faculty of Medicine, Yamaguchi University, Yamaguchi, Japan

**Keywords:** ANCA-associated vasculitis (AAV), ANCA, muscle involvement, proximal muscle weakness, truncal muscle weakness

## Abstract

**Introduction:**

Antineutrophil cytoplasmic antibody (ANCA)-associated vasculitis (AAV) frequently leads to mononeuritis multiplex, which are characterized by distal weakness associated with sensory disturbances. Although AAV has also been reported to be associated with myopathy, the pathogenesis and characteristics remain unclear. We aimed to show the clinical and laboratory findings in AAV-associated myopathy.

**Methods:**

This retrospective single-center study included patients with the diagnosis of AAV who had been admitted to the neurology department and had biopsy specimens of muscle and/or nerve tissue.

**Results:**

We identified four patients with a distinct clinical presentation of muscle weakness in the trunk and proximal limbs. The weakness resembled that of inflammatory muscle disease. These patients denied symptoms associated with neuropathy, and had normal serum creatine kinase (CK) levels. Needle electromyography (needle EMG) showed spontaneous electrical activity at rest, and results of magnetic resonance imaging (MRI) suggested inflammatory myopathy. Muscle biopsy specimens from all four patients revealed vasculitis and inflammation in proximity to the affected vessels, without any discernible characteristics of other myopathies. The patients also complained of symptoms affecting other organs, such as the ears and kidneys, which is typical of AAV cases. Remission induction therapy, such as cyclophosphamide pulse therapy in addition to oral prednisolone, were effective for all four patients. However, relapses occurred in two patients during maintenance therapy and two patients died of aspiration pneumonia.

**Discussion:**

The clinical course of our patients might represent a subtype of AAV that is characterized by muscle weakness of the trunk and proximal extremities and arises from vasculitis within the muscles. The clinical manifestations of our patients were similar to those of patients with inflammatory myopathy with regard to the distribution of muscle weakness, MRI and needle EMG findings. However, there are notable differences between AAV associated myopathy vs. inflammatory myositis like dermatomyositis; (1) the absence of elevated CK levels, (2) the presence of complications in other organs, (3) distinct pathological findings, and (4) severe outcomes. Awareness that AAV patients with muscle involvement could have a subtype of AAV that seriously affects various organs is critical for an accurate diagnosis and effective therapeutic management.

## Introduction

Antineutrophil cytoplasmic antibody (ANCA)-associated vasculitis (AAV) is a disease characterized by inflammation of the small blood vessels (1, 2). In AAV, vasculitis-induced ischemia, at least partially, results in organ damage (1, 2). AAV generally leads to deterioration of health-related quality of life because of damage to multiple organs, which in some cases is life-threatening (3–5).

The major clinicopathological subtypes of AAV are granulomatosis with polyangiitis (GPA), microscopic polyangiitis (MPA), and eosinophilic granulomatosis with polyangiitis (EGPA) (2). AAV affects various organs, including the ENTs (ears, nose, and throat), lungs, kidneys, skin, cardiovascular and gastrointestinal systems, and mucosa; as well as the central and peripheral nervous systems (6). Distal sensory disturbances and muscle weakness are common signs/symptoms of patients with AAV and peripheral nerve involvement. However, there have been several case reports of patients with AAV who present with weakness predominantly in the proximal extremities but do not show sensory deficits, which suggests that muscles are being affected (7–12). To our best knowledge, all of these published reports are of individual cases, and comprehensive details regarding the clinical and laboratory characteristics, response to treatment, and patient outcomes remain unknown.

For this study, we conducted a retrospective review of medical records at a single neurology department to identify patients with AAV and weakness predominantly affecting the trunk and proximal muscles. Here we report the neurological, serological, neurophysiological, pathological, and therapeutic features in detail of four patients with AAV and truncal muscle weakness that resembled an inflammatory myopathy.

## Materials and methods

### Ethics

This study was approved by the Institutional Review Board of the Yamaguchi University Hospital, Yamaguchi, Japan (approval number: 2022-227).

### Patients

The medical records of consecutive patients with the diagnosis of AAV who had been admitted to the Department of Neurology, Yamaguchi University Hospital between 2017 to 2022 were reviewed to identify patients who exhibited muscle weakness predominantly affecting the muscles of the trunk and proximal limbs. The diagnosis of AAV was based on the guidelines from the International Chapel Hill Consensus Conference (CHCC2012) (2).

In this study, weakness of the neck (flexors/extensors) and/or pectoralis major muscles was defined as truncal muscle weakness. Weakness of the deltoid, biceps brachii, triceps brachii, iliopsoas, quadriceps femoris, and hamstring muscles was defined as proximal muscle weakness; whereas weakness of the wrist flexor, wrist extensor, gastrocnemius, tibialis anterior, extensor hallucis longus, and flexor hallucis longus muscles was defined as distal muscle weakness.

### Clinical and laboratory findings

Clinical information included the following: age at onset; sex; manual muscle test (MMT) results during the initial visit; results of routine blood tests; serum ANCA antibody titers; levels of creatine kinase (CK), myoglobulin, and aldolase; information on other affected organs, treatments, and clinical course.

The assessments of weakness and other neurological findings were performed by two or more board-certified neurologists from the Department of Neurology at Yamaguchi University Hospital. ANCA antibody levels were measured by a chemiluminescent enzyme immunoassay. We evaluated the findings from muscle magnetic resonance imaging (MRI), Needle electromyography (needle EMG), and nerve conduction studies (NCS).

### Muscle biopsy

The site for the muscle biopsy was selected based on the findings of a neurological evaluation, muscle MRI, and needle EMG. Muscle specimens were placed on a flat piece of cork with gum tragacanth and fixed for 60 s in isopentane cooled by liquid nitrogen. Each specimen was cut into 10-μm sections, which were treated by hematoxylin-and-eosin and elastin-van Gieson stains, and immunohistochemical stains for the human major histocompatibility complex (MHC) class I (HLA-ABC) antigens. Pathological findings such as the presence of inflammatory cells in vessel walls, fibrinoid necrosis, endothelial disruption, and fragmentation of the internal elastic lamina were used to determine the presence of vasculitis.

## Results

### Patients

Between 2017 and 2022, a total of 21 patients with the diagnosis of AAV that was based on the criteria outlined in CHCC2012 underwent biopsies of either nerve and/or muscle tissue in the Department of Neurology. Among these 21 patients, four (2 men and 2 women) aged between 73 and 78 years presented with truncal and proximal muscle weakness (Table 1). Importantly, none of the four patients were aware of muscle weakness, especially of the neck flexors, although muscle weakness of the trunk muscles was obvious on examination. Muscle pain were observed from the onset of the disease in three of four patients.

**Table 1:** Clinical characteristics of 4 AAV patients with predominant truncal muscle weakness.

		Patient 1	Patient 2	Patient 3	Patient 4
Age at onset		77	78	77	73
Sex		Female	Male	Male	Female
First symptom		Muscle pains	Muscle weakness	Ear fullness	Muscle pains
Symptoms before ANCA measurement		Muscle pains, occult blood	Weakness	Ear fullness, occult blood	Muscle pains, preceding otitis
Lung involvement		−	−	−	−
The reason for neurologist’s consultation		Muscle pains	Muscle weakness	Muscle pains	Muscle pains
Rheumatology evaluation		+	−	+	+
Muscle pains		+	−	+	+
Muscle atrophy		−	−	−	−
Cranial nerve palsy		−	IX, X	−	−
Type of ANCA		MPO-ANCA	PR3-ANCA	MPO-ANCA	MPO-ANCA
ANCA titer (U/mL)		42.6	17.8	24.4	34.0
CK before therapy (U/L)		13	28	34	19
nEMG	Spontaneous discharge	+	+	+	−
	Early recruitment	+	+	+	−
	Myopathic units	+	−	+	−
T1WI isointense and T2WI hyperintense lesion of muscle on MRI		+	+	+	+
Differential diagnosis		AAV, IM	AAV, ALS, IM	AAV, IM	AAV, IM
Pathology	Marked infiltrate surrounding perimysium	+	+	+	+
	Fibrinoid necrosis	+	−	+	+
	Fragmentation of internal elastic lamina	−	−	−	+
Other organ involvement		RPGN	Stroke	RPGN, otitis	Otitis
Therapy	Remission induction	IVMP + PSL	PSL	IVCY + PSL	IVMP + PSL + IVCY
	Maintenance	PSL	PSL	RTX + PSL	PSL + AZT → PSL + AZT + RTX

ANCA, anti-neutrophil cytoplasmic antibody; CK, creatine kinase; nEMGm needle electromyogram; MRI, magnetic resonance imaging; AAV, ANCA associated vasculitis; IM, inflammatory myopathy; ALS, amyotrophic lateral sclerosis; RPGN, rapidly progressive glomerular nephritis; IVMP, intravenous methylprednisolone pulse; PSL, oral prednisolone; IVCY, intravenous cyclophosphamide pulse; RTX, rituximab; AZT, azathioprine.

### Disease onset and clinical course

In Patient 1, proximal symmetric muscle pain and fever occurred after influenza vaccination. Three weeks later, she took examinations at the department of rheumatology in a local general hospital. Based on the results for microscopic hematuria and positive serum myeloperoxidase (MPO)-ANCA, she was strongly suspected as AAV. She subsequently sought consultation with our neurology department to evaluate her muscle pain 6 weeks after the onset of symptoms. Patient 2 was referred to our neurology department from a local general hospital due to a 6-month history of difficulty walking caused by proximal muscle weakness. Patient 3 had refractory otitis and proximal muscle pain persisting for 1 month. He was referred to the department of Otorhinolaryngology at our hospital for examination of refractory otitis. Elevated serum inflammation markers led him seek an evaluation at the department of rheumatology in our hospital. He was strongly suspected as AAV based on microscopic hematuria, proteinuria, and positivity for MPO-ANCA result. He was referred to our neurology department to evaluate proximal muscle pain. Patient 4 was referred to our neurology department for an examination due to proximal and distal muscle pain persisting for 1–2 weeks, along with concurrent refractory otitis.

### Findings of neurological examinations

Muscle weakness of the trunk and proximal limb muscles of four patients is described in Table 2. None of the four patients exhibited distal limb weakness, which is a typical manifestation of the mononeuritis multiplex caused by AAV. Atrophic muscles were not observed. Although spontaneous muscle pain and muscle tenderness were observed in three patients, none of the patients exhibited sensory disturbances. Hyporeflexia of the lower limbs was seen in three of four patients. Patient 2 had an ischemic stroke and displayed hyper-reflexia of all the limbs, and a positive Babinski sign bilaterally. In Patient 2, mild ninth and tenth cranial nerve palsy were occasionally detected by neurologist when Patient 2 was referred to our neurology department.

**Table 2:** Manual muscle testing at first visit (right/left).

		Patient 1	Patient 2	Patient 3	Patient 4
Trunk muscles	Neck flexors	2	4	3	3
	Neck extensors	5	5	5	5
	Pectoralis major	4/4	5/5	5/5	4/4
Limb proximal muscles	Deltoid	5/5	5/5	5/5	5/5
	Biceps brachii	5/5	5/5	5/5	5/5
	Triceps brachii	5/5	4/4	5/5	5/5
	Iliopsoas	4/4	4/4	4/4	4/4
	Quadriceps femoris	5/5	5/5	5/5	4/4
	Hamstrings	4/4	4/4	4/4	4/4
Limb distal muscles	Wrist flexors	5/5	5/5	5/5	5/5
	Wrist extensors	5/5	5/4	5/5	5/5
	Gastrocnemius	5/5	5/5	5/5	5/5
	Tibialis anterior	5/5	5/5	5/5	5/5
	Extensor hallucis longus	5/5	5/5	5/5	5/5
	Flexor hallucis longus	NA	5/5	NA	5/5

NA, not available. Patient number accord with those in Table 1.

### Other affected organs

During their initial visit, two patients presented with mild rapidly progressive glomerular nephritis (RPGN) that did not require apheresis. Otitis was previously documented in two patients. Patient 2 presented with cerebral ischemia and underwent endovascular therapy. There was no evidence of malignancy or specific rash such as mechanic’s hands and Gottron’s sign in any of the patients, indicating the absence of complications typically associated with inflammatory myopathies.

### Laboratory findings

Serum creatine kinase levels ranged from 13 to 34 U/L and were within the normal range for all patients. Aldolase and myoglobin levels were also normal in every patient. Patients 1, 3, 4 were positive for MPO-ANCA, and Patient 2 was positive for proteinase (PR3)-ANCA. Autoantibodies associated with inflammatory myopathies, including anti-amino acyl-tRNA synthetase antibody, TIF1-γ antibody, MDA-5 antibody, and Mi-2 antibody, were not detected.

### Imaging and neurological test findings

**Figure 1:**
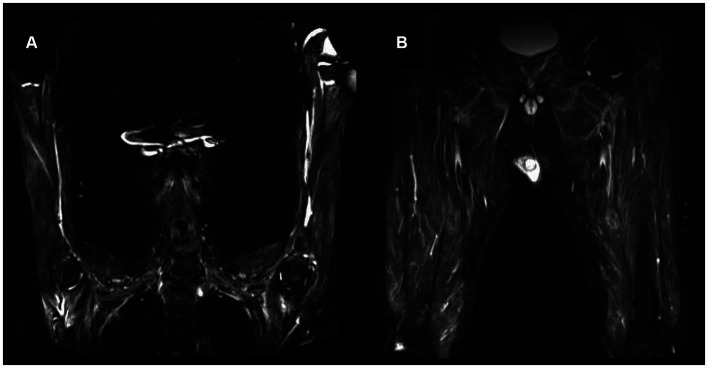
Magnetic resonance imaging (MRI) findings on short-tau inversion recovery (STIR) of proximal upper limb **(A)** and lower limb **(B)** in Patient 3. Muscle MRI demonstrated hyperintensity on STIR in muscle and connective tissue surrounding the muscle fasciae of proximal upper and lower limb.

Needle EMG revealed fibrillation potentials and positive sharp waves in three (75%) in four patients (Table 1). Myopathic units were confirmed in two patients (50%). Muscle MRI demonstrated hyperintensity on T2-weighted imaging and short-tau inversion recovery, with isointensity on T1-weighted imaging, in the muscles and connective tissues surrounding the muscle fasciae in three patients. These hyperintense lesions in muscle MRI were symmetrical and homogenous rather than patchy within muscles (Figure 1). None of the patients manifested the symptom of mononeuritis multiplex, although nerve conduction studies revealed decreased action potential amplitudes, suggesting asymptomatic mononeuritis multiplex in three of four patients.

### Histopathological findings

We conducted biopsies for diagnosing based on pathological findings in all patients. Patients 1 and 3 were strongly suspected to have AAV due to renal involvement and ANCA positivity; however, they did not receive any biopsy from other tissues. Consequently, we performed muscle biopsies. In Patients 2, 4, there are several potential differential diagnosis, and biopsy proved to be instrumental in arriving at a final diagnosis for AAV. Patient 2 presented with progressive muscle weakness and pyramidal sign, raising the possibility of amyotrophic lateral sclerosis or inflammatory myopathy, in addition to stroke as potential differential diagnosis. Patient 4 was suspected to have either AAV or inflammatory myopathy.

**Figure 2:**
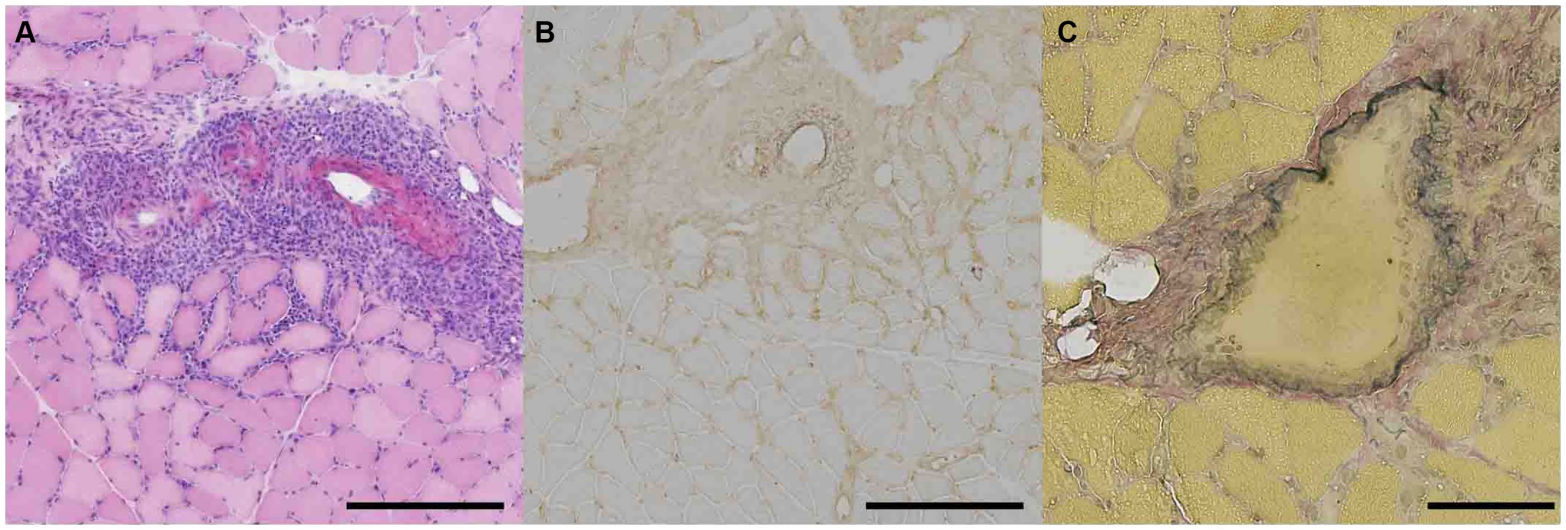
Pathological features of gastrocnemius muscle in patient 4 with ANCA associated vasculitis presenting trunk and proximal muscle weakness. **(A)** Hematoxylin and eosin staining of muscle fixed in isopentane cooled with liquid nitrogen was shown. Representative staining of marked infiltrate surrounding perimysium was shown. Scale bar = 500 μm. **(B)** Immunohistostaining of HLA-ABC in frozen section was presented. Scale bar = 500 μm. **(C)** Elastin-van Gieson staining in frozen section was demonstrated. Scale bar = 100 μm.

Muscle biopsies were performed on the vastus lateralis muscles of Patients 1, 2, and 3, and on the gastrocnemius muscle of Patient 4. Fibrinoid necrosis was observed in the walls of small arteries (outer diameters 100–200 μm) within the perimysium in three patients (Supplementary Figure S1). Marked inflammatory infiltrates surrounding the perimysium were observed in all four patients (Figure 2A). Immunohistochemical staining revealed highly expressed HLA-ABC antigens near the inflamed vessels in three patients (Figure 2B). Elastin-van Gieson staining demonstrated fragmentation of the internal elastic lamina in one patient (Figure 2C). The biopsy specimens were negative for the presence of perifascicular atrophy, perifascicular necrosis, muscle fiber regeneration, inflammatory infiltration of non-necrotic muscle fibers, elevated expression of myxovirus-resistance protein, and C5b-9 deposits.

### Results of treatments

Intravenous cyclophosphamide pulse therapy (500–1,000 mg/m^2^ body surface area every 4 weeks) was administered to two of four patients to induce remission (Table 1). Intravenous methylprednisolone pulse therapy (1 g/day for 3 days) was administered to two patients. Oral prednisolone (initially 1 mg/kg/day, tapered gradually) was given to all patients.

Following the induction of remission, three patients showed partial improvement on MMT and resolution of their muscle pain. Patient 1 underwent repeat needle EMG, which documented disappearance of denervation potentials after induction therapy. The other affected organs (RPGN and otitis) also showed improvement in Patients 1, 2, and 3.

All four patients received prednisolone orally as maintenance therapy. Two patients also received rituximab (375 mg/m^2^ body surface area every 6 months). Relapses of AAV occurred in two patients (Patients 1 and 4). Patient 1 developed a relapse of AAV associated with the development of interstitial pneumonia confirmed by elevated serum KL-6, ground-glass appearance in computed tomography while receiving oral prednisolone (7 mg/day). Patient 4 developed a relapse of AAV associated with development of cranial nerve palsies and an elevated erythrocyte sedimentation rate while receiving oral prednisolone at a dose of 12 mg/day and azathioprine at a dose of 75 mg/day. Patients 1 and 2 died of aspiration pneumonia while undergoing maintenance therapy.

## Discussion

In this study, we summarized the clinical and laboratory findings of four patients with AAV who exhibited weakness predominantly affecting their trunk and proximal muscles. While weakness predominantly involving the neck flexors and pectoralis major muscle is typically observed in inflammatory myopathy, it is rarely reported in AAV patients. We propose that the manifestations of our patients represent a subtype of AAV that is characterized by muscle weakness of the trunk and proximal extremities as a result of vasculitis within the muscles.

Six previous cases of AAV have been reported that manifested weakness predominantly affecting the proximal muscles (Table 3) (7–12), although these reports did not emphasize that their muscle weakness was localized. Importantly, all four of our patients did not report truncal muscle weakness, highlighting the possibility that muscle involvement in patients with AAV could be underestimated. In our institution, these patients accounted for 19% (four out of 21 patients) of biopsy-proven patients with AAV. Physician awareness that AAV patients can present as weakness of the trunk muscles is essential, because this condition is accompanied by distinct complications and requires unique therapeutic strategies.

**Table 3:** Reported cases of AAV who have trunk and proximal muscle weakness.

Authors	Age/gender	Diagnosis	Type of ANCA	Serum CK	Muscle biopsy	Other involved organs	Treatment
Birnbaum (2007)	77, female	MPA	MPO	Normal	Examined	Lung	IVCY
Bahou (2017)	63, female	MPA	MPO	367 U/L	Examined	Lung, kidney	IVMP
Ojima (2018)	58, male	MPA	PR3	134 U/L	Examined	None	IVMP + IVCY
Oiwa (2018)	76, female	AAV	MPO	25 U/L	Examined	Ear	PSL
Nagiah (2020)	82, male	AAV	MPO	211 U/L	Examined	None	PSL
Dutcher (2021)	69, female	GPA	PR3	Normal	Not examined	CNS, kidney	IVMP + RTX

AAV, ANCA-associated vasculitis; CK, creatine kinase; MPA, microscopic polyangiitis; GPA, granulomatosis with polyangiitis; MPO, myeloperoxidase; PR3, proteinase 3; CNS, central nervous system; IVCY, intravenous cyclophosphamide; IVMP, intravenous methylprednisolone; RTX, rituximab.

Although other affected tissues and organs varied among our patients, which included the ears (otitis), lungs (alveolar hemorrhage), kidneys (acute injury), and brain (cerebral ischemia), all of these complications were typical of AAV, whereas inflammation of the muscles is not. Previous AAV associated myopathy cases (Table 3) also showed other affected organs typical for AAV. In addition to these reports, there is a reported patients with AAV who presented biopsy-proven vasculopathy in gastrocnemius muscle with no description of muscle weakness (13), which further confirm that AAV associated myopathy patients have complications similar to the typical AAV cases.

Although distribution of muscle weakness between AAV associated myopathy, inflammatory myopathy, and other connective tissue disease such as systemic sclerosis are similar, the therapeutic managements are different in each disease (14, 15). Patients with inflammatory myopathy usually are treated by steroids and mild immunosuppressants such as azathioprine (14). Fibrosing myopathy in systemic sclerosis are resistance for immunotherapy because of absence of inflammation within muscle (15). AAV associated myopathy patients generally require immunotherapies stronger than azathioprine. The American College of Rheumatology/Vasculitis Foundation Guidelines recommend rituximab or cyclophosphamide as the initial treatment for patients with severe active GPA or MPA (16). Severe GPA and MPA are defined as vasculitis with life- or organ-threatening manifestations (e.g., alveolar hemorrhage, glomerulonephritis, central nervous system vasculitis, mononeuritis multiplex, cardiac involvement, mesenteric ischemia, limb/digit ischemia). Muscle problems are not included in the definition. Therefore, according to the guidelines, patients with AAV and muscle weakness only should be treated by methotrexate and glucocorticoids according to this guideline (16).

Most of our study patients required strong remission induction therapies such as cyclophosphamide or intravenous methylprednisolone pulse therapy followed by high-dose oral prednisolone. Furthermore, clinical relapses occurred during maintenance therapy in half of our patients, suggesting the need for prolonged maintenance therapies such as rituximab, even for patients with AAV presenting with muscle problems without severe involvement of other organs.

Ojima et al. reported similar findings. They reported a patient with AAV and isolated muscle weakness who required aggressive immunotherapy (10). Altogether, our findings and those of Ojima et al. suggest that AAV-associated myopathy should be considered a condition distinct from inflammatory myopathy with regard to responses to treatment and outcomes. In patients who are suspected to have inflammatory myopathy based on their neurological findings, AAV-associated myopathy should be included in the differential diagnosis, even in the absence of the involvement of other organs.

AAV patients with muscle involvement who underwent muscle MRI exhibited hyperintensity on T2-weighted imaging and spontaneous electrical activity at rest on needle EMG. These findings are typical of those seen in patients with inflammatory myopathy. However, the serum CK, myoglobin, and aldolase levels were normal in our patients, suggesting that the main immunological target is not the affected muscle. We confirmed this by the direct detection of vasculitis in muscle biopsy specimens from these patients. Histopathological studies also confirmed the upregulation of HLA-ABC antigen expression in the muscle fibers surrounding the inflamed vessels, which suggests that inflammation primarily occurs near the vessels. These findings led us to conclude that the MRI and needle EMG findings in our patients merely reflect secondary inflammation of the muscles.

In conclusion, our study suggests that in some patients with AAV, weakness predominantly manifested by the muscles of the trunk and proximal limbs is caused by the vasculitis. This subtype of AAV is characterized by normal serum CK levels, the presence of ANCA, and involvement of other organs, similar to typical AAV. Strong immunotherapies might be necessary for the treatment of patients with this subtype. Awareness that weakness of the muscles of the trunk and proximal limbs can be a manifestation of AAV is crucial for an accurate diagnosis and the selection of appropriate immunotherapy as the first-line treatment.

## Data availability statement

The raw data supporting the conclusions of this article will be made available by the authors, without undue reservation.

## Ethics statement

The studies involving humans were approved by Institutional Review Board of Yamaguchi University Hospital. The studies were conducted in accordance with the local legislation and institutional requirements. Written informed consent for participation was not required from the participants or the participants’ legal guardians/next of kin because written informed consent was not required in accordance with the national legislation and the institutional requirements.

## Author contributions

JN: Writing – original draft. HN: Writing – original draft. TY: Writing – original draft. RS: Writing – review & editing, Data curation. MK: Writing – review & editing. TK: Writing – review & editing. MN: Writing – review & editing. TY and RS: Data curation.
