# Supplementary material for: Antineutrophil cytoplasmic antibody-associated vasculitis with predominant truncal muscle weakness: a retrospective case series
Source: Front Neurol. 2023 Oct 12;14:1277337. doi: 10.3389/fneur.2023.1277337 (PMC10603186; doi:10.3389/fneur.2023.1277337)
Supplement: Supplementary file 1 [file Image_1.pdf]

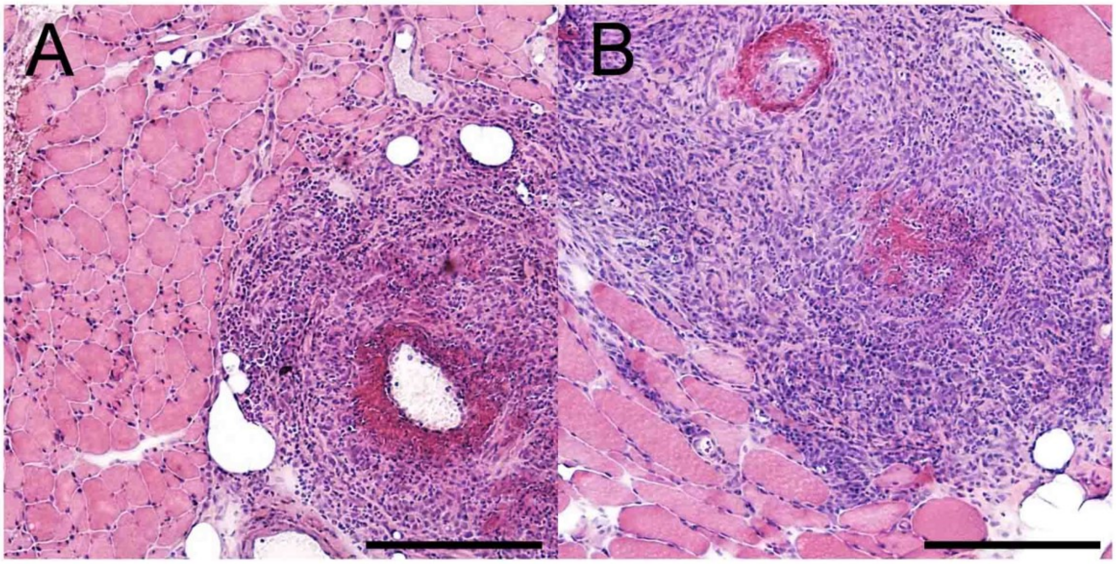

Figure S1 Pathological features in Patients 1, 3 with ANCA associated vasculitis presenting trunk and proximal muscle weakness. Hematoxylin and eosin staining of vastus lateralis muscle in Patient 1 (A) and Patient 3 (B) were demonstrated. Marked infiltrate surrounding perimysium and fibrinoid necrosis were observed in both cases. We only obtained evidence of infiltrate in Patient 2 (data not shown). Scale bar = 500 μm.
